# SFE-GAT: Structure-Feature Evolution Graph Attention Network for Motor Imagery Decoding

**DOI:** 10.3390/s26051730

**Published:** 2026-03-09

**Authors:** Xin Gao, Guohua Cao, Guoqing Ma

**Affiliations:** 1 School of Mechatronic Engineering, Changchun University of Science and Technology, Changchun 130022, China; 2 Chongqing Research Institute, Changchun University of Science and Technology, Chongqing 401133, China

**Keywords:** motor imagery EEG, graph neural network, brain network dynamics, functional connectivity

## Abstract

Motor imagery EEG decoding often relies on static functional connectivity graphs that cannot capture the dynamic, stage-wise reorganization of brain networks during tasks. This paper aims to develop a graph neural network that explicitly simulates this neurodynamic process to improve decoding and provide computational insights. This paper proposes a Structure-Feature Evolution Graph Attention Network (SFE-GAT). Its inter-layer evolution mechanism dynamically co-adapts graph topology and node features, mimicking functional network reorganization. Initialized with phase-locking value connectivity and spectral features, the model uses a graph autoencoder with Monte Carlo sampling to iteratively refine edges and embeddings. On the BCI Competition IV-2a dataset, SFE-GAT achieved 77.70% (subject-dependent) and 66.59% (subject-independent) accuracy, outperforming baselines. Evolved graphs showed sparsification and strengthening of task-critical connections, indicating hierarchical processing. This paper advances EEG decoding through a dynamic graph architecture, providing a computational framework for studying the hierarchical organization of motor cortex activity and linking adaptive graph learning with neural dynamics.

## 1. Introduction

Brain–Computer Interfaces (BCIs) enable direct interaction between the brain and external devices by decoding signals such as electroencephalography (EEG) [1]. Among BCI paradigms, motor imagery (MI) is particularly attractive for rehabilitation and control applications because it elicits task-specific EEG patterns without overt movement [2,3]. However, MI-EEG decoding remains challenging due to the signals’ low signal-to-noise ratio, pronounced intersubject variability, nonstationarity, and the distributed nature of task-relevant information across dynamically interacting brain regions [4,5,6]. Accordingly, effectively leveraging the spatial–topological structure of multichannel EEG is critical for improving decoding performance [7,8].

In recent years, graph neural networks (GNNs) have shown substantial promise for this task. By representing multichannel EEG as functional brain connectivity graphs, GNNs directly model inter-regional coordination patterns, providing a principled route to accurate MI intent recognition [9,10,11,12].

A wide range of GNN-based methods for MI-EEG decoding has been explored [13,14]. Some construct static connectivity graphs using neuroanatomical priors or measures such as the phase-locking value (PLV) [9,10]. Others learn the graph structure to reduce reliance on fixed priors [15,16]. Despite increased flexibility, the learned graphs are typically global or fixed at the input layer, rather than evolving through the feature-extraction hierarchy [17]. In effect, these approaches still assume a static or quasi-static topology that is shared across GNN layers [18]. Neuroscientific studies indicate that motor imagery entails a dynamic, hierarchical reconfiguration of functional brain connectivity, progressing from localized sensorimotor regions to globally integrated networks [19]. However, existing GNN approaches based on static graphs are limited in their ability to capture the depth-dependent evolution of functional topology across stages of cognitive processing, which can constrain decoding performance [20,21].

Therefore, this study proposes a dynamic graph neural network with a layerwise graph evolution mechanism, termed SFE-GAT. The mechanism is designed to simulate dynamic adjustments of functional brain connections during task execution. These adjustments are modeled as a function of the processing stage determined by the network hierarchy. Shallow layers are expected to capture local and stable connectivity patterns. Deep layers are expected to evolve global and abstract connectivity patterns relevant to task decision making.

This model does not treat the functional brain connectivity graph as fixed but rather as an entity that can evolve dynamically across network layers. Specifically, within each graph attention layer, the model alternately optimizes node features and graph topology: it employs a Graph Attention Network to aggregate node information while simultaneously using a lightweight Graph Autoencoder to dynamically update the inter-layer adjacency relationships based on the feature representations learned at the current layer. This co-evolution process enables the model to adaptively learn functional connectivity patterns at different abstraction levels for shallow and deep layers, thereby more accurately characterizing the dynamic brain activity underlying MI tasks [22,23].

The contributions of this study are threefold:

We introduce a inter-layer graph-evolution mechanism based on dynamic graph structure learning for MI-EEG decoding. This mechanism removes dependence on predefined static graphs and enables joint adaptation of graph topology and node representations across layers.

We develop a compact architecture that integrates a Graph Attention Network with a Graph Autoencoder. Through a differentiable sampling strategy, it dynamically updates the inter-layer graph structure, improving representational capacity while keeping the parameter budget comparable to conventional GNNs that use static graphs.

Extensive experiments on the BCI Competition IV-2a dataset show that SFE-GAT outperforms a range of baseline models in subject-dependent and subject-independent settings, demonstrating its effectiveness. Visual analyses of the learned graphs further offer insight into motor-imagery-related brain dynamics.

## 2. Materials and Methods

**Figure 1:**
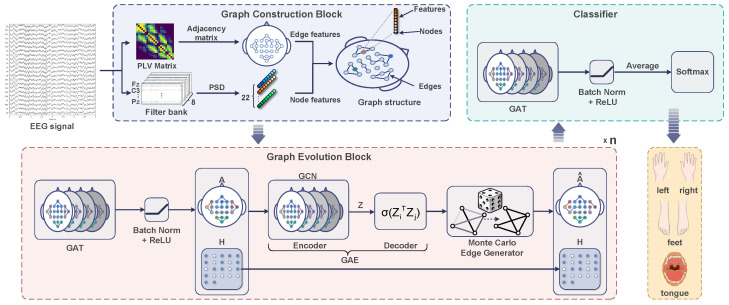
The SFE-GAT framework primarily involves three steps: constructing the initial graph structure and node features using PLV and PSD; evolving the graph topology and features based on GAT and GAE; and producing the final output via the classifier.

The framework of the proposed SFE-GAT, illustrated in Figure 1, comprises three core modules: graph construction, graph evolution, and a classifier. The graph construction module first defines inter-node connectivity based on the Phase-Locking Value (PLV) and defines spectral band Power Spectral Density (PSD) as node features, thereby transforming the raw MI-EEG signals into graph-structured data. Subsequently, the graph evolution module dynamically updates the graph topology through iterative operations involving graph attention, batch normalization, and graph autoencoding. Finally, the classifier decodes the evolved node features and accomplishes motor imagery task recognition using a fully connected layer followed by a softmax classifier.

The three core blocks of the SFE-GAT are elaborated in detail in the following subsections. The graph construction block transforms raw EEG-MI time-series signals into graph-structured data.

### 2.1. Graph Construction Block

The graph construction module aims to transform raw neural signal data into a graph structure, providing the foundation for subsequent graph neural network modeling. The Phase-Locking Value (PLV) is employed to compute the functional connectivity between nodes (electrodes), thereby analyzing the brain’s functional networks. PLV measures the phase synchronization between two signals by assessing whether their phase difference remains relatively constant over time, reflecting the degree of temporal phase locking [24]. PLV is sensitive to instantaneous phase changes in EEG signals and can provide information about the coupling between different brain regions [25].

To compute the PLV, the instantaneous phase $\theta_{i}\left(t\right)$ is first extracted for each electrode’s signal via the Hilbert transform. For any electrode pair (i,j), the instantaneous phase difference is $\Delta \theta_{ij}\left(t\right)=\theta_{i}\left(t\right)-\theta_{j}\left(t\right)$. The PLV is then calculated as the magnitude of the average of the unit complex vectors corresponding to this phase difference over time:

(1) $${\mathrm{PLV}}_{ij}=\left|\frac{1}{T}\sum_{t=1}^{T}e^{i\Delta \theta_{ij}\left(t\right)}\right|$$

where *T* is the total number of time points, and $e^{i\Delta \theta_{ij}\left(t\right)}$ is the unit vector representation of the phase difference $\Delta \theta_{ij}\left(t\right)$ in the complex plane. The value of PLV ranges between [0,1], with values closer to 1 indicating stronger phase synchronization between electrodes *i* and *j*.

Based on the computed Phase-Locking Value matrix $\mathrm{PLV}\in R^{C\times C}$ (where *C* is the number of electrodes), the final adjacency matrix A, which defines the edge connections of the graph structure, is constructed through the following sequential steps.

First, to eliminate global scale variations, the original PLV matrix is standardized. Specifically, the mean μ and standard deviation σ of all elements in the matrix are calculated as follows:

(2) $$\begin{array}{cc} \mu & =\frac{1}{C^{2}}\sum_{i=1}^{C}\sum_{j=1}^{C}PLV_{ij}^{\prime }\\ \sigma & =\sqrt{\frac{1}{C^{2}}\sum_{i=1}^{C}\sum_{j=1}^{C}{\left(PLV_{ij}^{\prime }-\mu \right)}^{2}} \end{array}$$

Each element is then standardized using the following equation:

(3) $$PLV_{ij}^{\prime }=\frac{P_{ij}-\mu }{\sigma +\epsilon }$$

where ϵ is a small constant used to ensure numerical stability. We set $\epsilon =1\times 10^{-8}$ to ensure numerical stability, following common practices in z-score standardization to prevent division by near-zero standard deviation.

Next, to prevent self-connections in the graph structure, the diagonal elements of the standardized matrix ${\mathrm{PLV}}^{\prime }$ are set to zero:

(4) $$PLV_{ii}^{\prime }=0,\;\forall i\in \left[1,C\right]$$

Finally, to construct a sparse adjacency matrix that retains only significant functional connections, a threshold τ is applied for filtering. An edge is retained only if the standardized value $PLV_{ij}^{\prime }$ is greater than τ, resulting in the final adjacency matrix A, whose elements are defined as:

(5) $$A_{ij}=\left\{\begin{array}{cc} PLV_{ij}^{\prime }, & \mathrm{if}\;PLV_{ij}^{\prime }>\tau \\ 0, & \mathrm{otherwise} \end{array}\right.$$

To construct a complete graph-structured dataset, this module further generates a feature representation for each node (electrode), building upon the defined edge connections (adjacency matrix). The node features are derived from the power spectral density (PSD) of specific frequency bands to capture rhythm activities associated with motor imagery tasks [26].

Specifically, the EEG signal x(t)from each electrode is first bandpass-filtered within the [8, 40] Hz range using a 4 Hz step size, yielding a series of sub-band signals:

(6) $$x_{k}\left(t\right)=\mathrm{Bandpass}\left(x\left(t\right),f_{\mathrm{low}}^{k},f_{\mathrm{high}}^{k}\right)$$

The 4 Hz step size is chosen following established filter-bank approaches in motor imagery EEG decoding, such as the Filter Bank Common Spatial Pattern (FBCSP) method [27,28], which balances spectral resolution and computational efficiency while capturing fine-grained differences in the μ (8–12 Hz) and β (13–30 Hz) sub-bands relevant to MI tasks. Bandpass filtering was performed using zero-phase 4th-order Butterworth filters (scipy.signal implementation) with passband ripple Rp = 0.5 dB and stopband attenuation Rs = 40 dB. The transition bandwidth is approximately determined by the filter order and the 250 Hz sampling rate.

For each filtered signal $x_{k}\left(t\right)$, its power spectral density $PSD_{k}\left(f\right)$, representing the signal’s power distribution at frequency *f*, is estimated using Welch’s method:

(7) $$PSD_{k}\left(f\right)=\frac{1}{T}{\left|\sum_{t=1}^{T}x_{k}\left(t\right)e^{-i2\pi ft}\right|}^{2}$$

Here, *T* denotes the signal duration, *f* represents frequency, and $x_{k}\left(t\right)$ is the bandpass-filtered signal. Welch’s PSD was estimated with 1-second segments (*T* = 250 samples) using 50% overlap and a Hann window to balance frequency resolution and adherence to local stationarity in MI-EEG signals. The total power within the sub-band is then computed as the feature value via integration:

(8) $$PSD_{k}^{band}=\int_{f_{\mathrm{low}}^{k}}^{f_{\mathrm{high}}^{k}}PSD_{k}\left(f\right)\;df$$

This process is repeated for all *B* sub-bands, generating a feature vector $h_{i}={\left[PSD_{1}^{band},PSD_{2}^{band},\ldots ,PSD_{B}^{band}\right]}^{⊤}\in R^{B}$ for each electrode *i*. Finally, the feature vectors from all electrodes are stacked to form the global node feature matrix $H={\left[h_{1},h_{2},\ldots ,h_{C}\right]}^{⊤}\in R^{C\times B}$, where *C* is the number of electrodes.

At this stage, the graph-structured data G=(V,E,H) is fully constructed: the node set V corresponds to the electrodes, the edge set E and its weights are defined by the aforementioned adjacency matrix A, which is based on the PLV, and the node features are provided by the frequency band power matrix H. This graph structure serves as the input for the subsequent graph evolution block.

### 2.2. Graph Evolution Block

The graph evolution block constitutes the core of this study, aiming to dynamically adjust the graph structure to achieve the co-optimization of node and edge relationships. The block adopts a multilayer architecture that alternates Graph Attention Network (GAT) and Graph Autoencoder (GAE) modules, enabling iterative updates of node features and graph topology across layers [29,30].

Specifically, we first apply GATv2 to aggregate neighborhood information from node features. GATv2 employs a dynamic attention mechanism that better captures complex inter-node dependencies [31]. It computes the attention coefficient $\alpha_{ij}$ between node *i* and each neighbor j∈N(i). This computation involves two steps: first, calculating the attention energy score for a node pair; second, normalizing this score. The detailed procedure is as follows.

First, the attention energy score $e_{ij}$ for the node pair (i,j) is calculated:

(9) $$e_{ij}=\mathrm{LeakyReLU}\left(a^{⊤}\left[Wh_{i}\|Wh_{j}\right]\right)$$

Here, W is a learnable weight matrix, a is the parameter vector of the attention mechanism, and ‖ denotes the vector concatenation operation. Subsequently, the Softmax function is applied to normalize the energy scores over the neighbor nodes *j*, yielding the final attention coefficient:

(10) $$\alpha_{ij}=\frac{\exp(e_{ij})}{\sum_{k\in N(i)}\exp\left(e_{ik}\right)}$$

After obtaining the attention coefficients, the updated feature representation for node *i* is generated by performing a weighted aggregation of its neighbors’ features, followed by a linear transformation and a nonlinear activation function:

(11) $$h_{i}^{\prime }=\sigma \left(W\sum_{j\in N(i)}\alpha_{ij}h_{j}\right)$$

After applying the above operation to all nodes in the graph, a new node feature matrix processed by one graph attention layer is obtained. The overall computation can be succinctly expressed as:

(12) $$H_{\mathrm{gat}}^{\left(\mathrm{evo}\right)}=\mathrm{ReLU}\left(\mathrm{GATv}2\left(H,A\right)\right)$$

To enhance the stability and convergence efficiency of the training process, batch normalization is applied after the graph attention layer. Batch normalization stabilizes the distribution of activations for each feature dimension. This primarily smooths the optimization landscape of the loss function, resulting in more stable gradients, permitting the use of larger learning rates, and thereby significantly improving convergence speed and training stability [32,33]. It is computed as follows:

(13) $$h_{:,j}^{\mathrm{bn}}=\gamma_{j}\cdot \left(\frac{h_{:,j}-\mu_{j}}{\sigma_{j}}\right)+\beta_{j}$$

Here, $h_{:,j}$ denotes the vector of all samples (in the batch) for the j-th feature dimension, while $\mu_{j}$ and $\sigma_{j}$ are the mean and standard deviation computed from the current mini-batch data for that feature dimension. $\gamma_{j}$ and $\beta_{j}$ are learnable scaling and shift parameters that preserve the model’s expressive power. The node feature matrix after batch normalization is denoted as:

(14) $$H_{\mathrm{bn}}^{\left(\mathrm{evo}\right)}=\mathrm{BatchNorm}\left(H_{\mathrm{gat}}^{\left(\mathrm{evo}\right)}\right)$$

Subsequently, the model utilizes a Graph Autoencoder (GAE) for graph structure reconstruction and representation learning. As an unsupervised learning model, the GAE aims to learn a low-dimensional, dense representation of the graph through an encoder-decoder framework [34]. Specifically, the encoder consists of a Graph Convolutional Network (GCN) layer that maps the normalized node features $H_{\mathrm{bn}}$ to a low-dimensional latent space representation $Z_{\mathrm{enc}}$:

(15) $$Z_{\mathrm{enc}}=\mathrm{ReLU}\left(\mathrm{GCNConv}(H_{\mathrm{bn}}^{\left(\mathrm{evo}\right)},A)\right)$$

The decoder then reconstructs the adjacency matrix via the inner product of the node latent representations $Z_{\mathrm{enc}}$. Specifically, the probability of a connection between node *i* and node *j* is given by the inner product of their feature vectors activated by the Sigmoid function:

(16) $$P_{\hat{A}}\left(i,j\right)=\sigma \left(z_{i}^{⊤}z_{j}\right)$$

where σ(·) is the Sigmoid function, mapping the inner product value to a probability within the [0,1] interval. This encoder–decoder framework drives the model to learn low-dimensional node representations that encapsulate the graph’s latent structure by reconstructing the graph topology.

To achieve adaptive adjustment of the graph structure during both training and evaluation phases, the model employs differentiated edge generation strategies. The mechanism generates a binary mask matrix M from the edge-probability matrix $P_{\hat{A}}$ produced by the graph autoencoder. The mask is then used to sparsify the original adjacency matrix, yielding the updated adjacency matrix $\hat{A}=A⊙M$, where ⊙ denotes the Hadamard product.

During training, stochasticity is introduced to enhance robustness and mitigate overfitting. Specifically, Monte Carlo sampling is implemented by comparing the edge probabilities with an i.i.d. uniform random matrix R∼U(0,1) of matching dimensions, thereby generating a dynamic mask:

(17) $$M=I(P_{\hat{A}}>R)$$

This operation is equivalent to independently sampling each edge with probability $P_{\hat{A}}\left(i,j\right)$, thereby inducing slight variations in the graph topology across training iterations and improving generalization.

In contrast, during evaluation, a deterministic strategy is adopted to ensure stable and reproducible performance assessment. The mask matrix is generated using a fixed threshold τ:

(18) $$M=I(P_{\hat{A}}>\tau )$$

This procedure ensures a deterministic graph structure during evaluation, thereby facilitating fair and reliable comparisons of model performance. This completes one cycle of co-update for the graph structure and node features within a single graph evolution block, which can be abstracted as the function:

(19) $$H^{\prime },A^{\prime }=\mathrm{GraphEvolutionLayer}\left(H,A\right)$$

By stacking L=3 such blocks, the model can capture hierarchical dynamic functional connectivity patterns, ultimately providing the classifier with the evolved high-level representations $H_{\mathrm{evolved}}$ and $A_{\mathrm{evolved}}$.

### 2.3. Classifier

The classifier aims to generate the final classification predictions based on the evolved graph structure $A_{\mathrm{evolved}}$ and node features $H_{\mathrm{evolved}}$. First, a Graph Attention Network (GATv2) layer is applied to perform a final round of information aggregation on the node features, fully leveraging the information from the evolved graph structure:

(20) $$H_{\mathrm{gat}}^{\left(\mathrm{cls}\right)}=\mathrm{GATv}2\left(H_{\mathrm{evolved}},A_{\mathrm{evolved}}\right)$$

Subsequently, batch normalization is applied to the aggregated features to stabilize the training process and accelerate convergence:

(21) $$H_{\mathrm{bn}}^{\left(\mathrm{cls}\right)}=\mathrm{BatchNorm}\left(H_{\mathrm{gat}}^{\left(\mathrm{cls}\right)}\right)$$

The result is denoted as the node feature representation used by the classifier: $H_{\mathrm{cls}}=H_{\mathrm{bn}}^{\left(\mathrm{cls}\right)}$. Global Mean Pooling is then employed to aggregate the node-level feature matrix $H_{\mathrm{cls}}\in R^{C\times D}$ into a graph-level representation vector $h_{\mathrm{graph}}\in R^{D}$:

(22) $$h_{\mathrm{graph}}=\frac{1}{C}\sum_{i=1}^{C}H_{\mathrm{cls}}\left[i,:\right]$$

where *C* is the number of nodes (electrodes). To enhance the model’s generalization capability, a Dropout layer with a rate of p=0.5 is applied for regularization after pooling:

(23) $$h_{\mathrm{drop}}=\mathrm{Dropout}\left(h_{\mathrm{graph}},p=0.5\right)$$

Finally, a fully connected layer maps the graph-level representation to the class space, and the Softmax function outputs a normalized categorical probability distribution:

(24) $$\hat{y}=\mathrm{Softmax}\left(W_{\mathrm{fc}}h_{\mathrm{drop}}+b_{\mathrm{fc}}\right)$$

Here, $W_{\mathrm{fc}}$ and $b_{\mathrm{fc}}$ are the weight matrix and bias vector of the fully connected layer.

## 3. Experiments and Results

### 3.1. Dataset and Processing

This study evaluates the proposed model on the publicly available BCI Competition IV-2a dataset [35].

**Figure 2:**
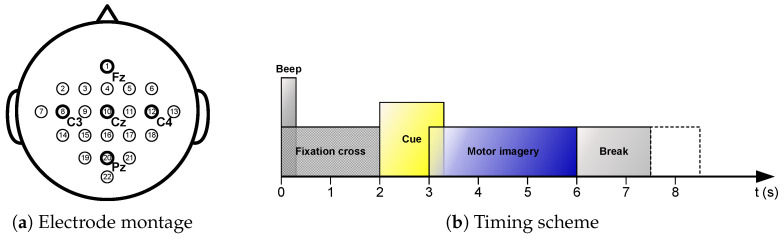
Description of the BCI Competition IV-2a dataset. (**a**) Electrode montage corresponding to the International 10–20 system. (**b**) Timing scheme of the experimental paradigm.

The dataset contains electroencephalogram (EEG) signals recorded from nine subjects performing four-class motor imagery tasks (left hand, right hand, both feet, and tongue). The data were recorded using 22 electrodes (arranged according to the International 10–20 system, see Figure 2a) at a sampling rate of 250 Hz, and were preprocessed with a 0.5–100 Hz bandpass filter followed by a 50 Hz notch filter.

The data for each subject comprises two sessions recorded on separate days, with each session containing 288 trials (i.e., 72 trials per class). The detailed timing scheme of a single trial, including the cue, preparation, task execution, and rest periods, is illustrated in Figure 2b. For subsequent analysis, EEG signals from all 22 channels during the task execution period (t = 2 s to t = 6 s) were extracted. No additional preprocessing, such as artifact removal, was applied to validate the model’s robustness on raw data. The data split follows the dataset’s predefined scheme, where the first session is used for model training and the second session for testing.

### 3.2. Performance Metrics

To comprehensively evaluate the proposed model, this study employs multiple metrics, including accuracy, precision, the F1-score, and the Kappa value.

(25) $$\mathrm{ACC}=\frac{\sum_{i=1}^{n}{\mathrm{TP}}_{i}/I_{i}}{n}$$

(26) $$\mathrm{Precision}=\frac{1}{n}\sum_{i=1}^{n}\frac{{\mathrm{TP}}_{i}}{{\mathrm{TP}}_{i}+{\mathrm{FP}}_{i}}$$

(27) $$F1=\frac{1}{n}\sum_{i=1}^{n}\frac{2\times {\mathrm{Precision}}_{i}\times {\mathrm{Recall}}_{i}}{{\mathrm{Precision}}_{i}+{\mathrm{Recall}}_{i}}$$

(28) $$\mathrm{Kappa}=\frac{P_{o}-P_{e}}{1-P_{e}}$$

Here, for class *i*: ${\mathrm{TP}}_{i}$ is the number of correctly predicted samples, ${\mathrm{FP}}_{i}$ is the number of false positives, and ${\mathrm{FN}}_{i}$ is the number of false negatives. $I_{i}$ is the total number of samples, and *n* denotes the number of classes. Additionally, $P_{o}$ represents the observed agreement, and $P_{e}$ represents the expected agreement. ${\mathrm{Precision}}_{i}$ and ${\mathrm{Recall}}_{i}$ denote the precision and recall for class *i*.

### 3.3. Experiment Setup

All experiments were conducted on a workstation equipped with an Intel(R) Xeon(R) Platinum 8358P CPU (2.60 GHz) and an NVIDIA GeForce RTX 3090 GPU. The operating system was Ubuntu 22.04, with CUDA version 12.4, Python version 3.10.16, and the deep learning frameworks PyTorch 2.7.0 and PyTorch Geometric 2.6.1. The proposed model contains approximately 0.15M trainable parameters.

The model was evaluated under both subject-dependent and subject-independent paradigms. In the subject-dependent paradigm, adhering to the original competition’s split, a session-level holdout method was employed: the 288 trials × 9 subjects from the first session were used as the training set, and the 288 trials × 9 subjects from the second session constituted the test set. The training and testing data were non-overlapping, and cross-validation was not performed. In the subject-independent paradigm, a leave-one-subject-out (LOSO) cross-validation was used for cross-subject evaluation. The number of folds equaled the number of subjects; in each fold, one subject was selected as the test set, while the remaining subjects were used for training, thereby assessing the model’s generalization capability on unseen subjects.

Furthermore, the parameter settings for the proposed model framework are detailed in Table 1. The model was trained using a Stochastic Gradient Descent (SGD) optimizer with a learning rate of 0.01, a batch size of 64, and a weight decay of 0.001. The loss function was categorical cross-entropy, and training proceeded for 500 epochs. These hyperparameters were selected based on preliminary experiments to balance convergence stability and generalization performance.

### 3.4. Experimental Results

**Figure 3:**
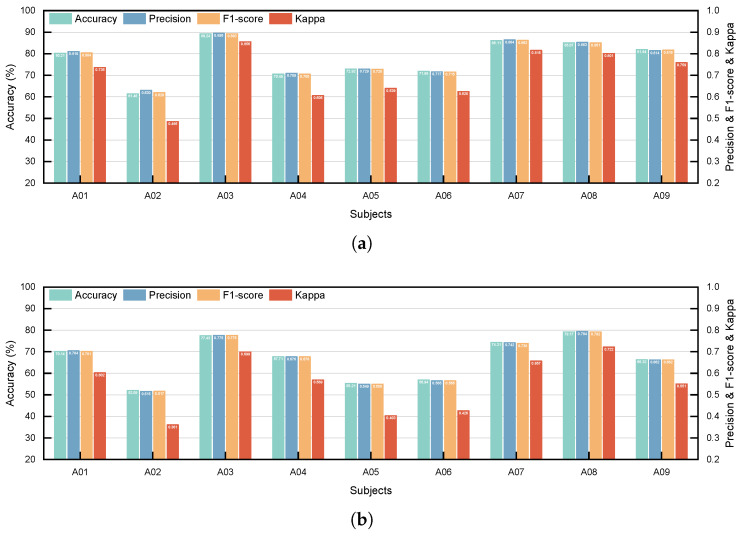
Performance of the proposed model under two learning paradigms. (**a**) HoldOut; (**b**) LOSO.

Figure 3 presents the overall performance of the proposed model under both subject-dependent (SD) and subject-independent (SI) evaluation paradigms. The model demonstrates strong performance across multiple evaluation metrics. Specifically, under the SD paradigm (see Figure 3a), significant results were achieved for all subjects except A02. Subject A03 exhibited the best performance, with accuracy, precision, F1-score, and Kappa coefficient values of 89.24%, 0.895, 0.893, and 0.856, respectively. In contrast, Subject A02 showed lower performance, with corresponding values of 61.46%, 0.630, 0.620, and 0.486. The lower performance for A02 may be related to individual differences, as the literature has also reported the existence of the “BCI-illiteracy” phenomenon in some subjects [36,37,38].

Under the SI paradigm (see Figure 3b), the model also performed excellently for the majority of subjects, again except for A02. Subject A08 achieved the best performance, with accuracy, precision, F1-score, and Kappa coefficient values of 79.17%, 0.794, 0.792, and 0.722, respectively. For Subject A02, the four metrics were 52.08%, 0.515, 0.517, and 0.361. These results indicate that the proposed model performs robustly under both evaluation paradigms, validating the effectiveness of its structural design and demonstrating its robustness across different evaluation settings.

### 3.5. Ablation Experiment

**Figure 4:**
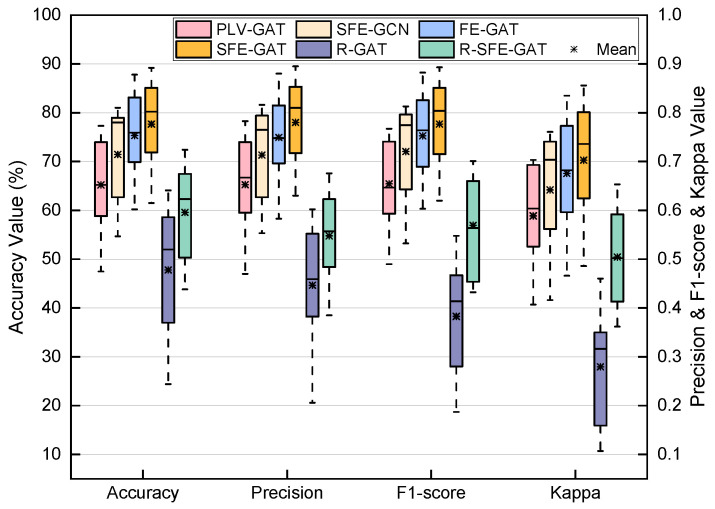
Ablation study results under the subject-dependent (SD) holdout paradigm. PLV-GAT denotes the model variant where the graph evolution module is removed, retaining only the PLV-based graph structure for classification with a GAT. SFE-GCN represents the variant where the graph attention mechanism is replaced with graph convolutional layers. FE-GAT indicates the variant where the edge evolution mechanism is removed, relying solely on the GAT-based feature evolution. R-GAT denotes the variant using a random adjacency matrix initialization without the graph evolution block, while R-SFE-GAT applies the full graph evolution mechanism starting from the same random initialization.

This section presents an ablation study to evaluate the effectiveness of the various mechanisms within the model. Figure 4 illustrates the impact of disabling or replacing specific mechanisms in the SFE-GAT on performance for the MI classification task using the BCI-2a dataset; all modifications were applied before the commencement of training and validation. Compared to the complete model, directly removing the graph evolution module resulted in an overall accuracy drop of 12.5%. Replacing the GAT with a GCN within the model, thereby removing the attention mechanism, led to a 6.25% decrease in accuracy. Removing the edge evolution mechanism based on the GAE and Monte Carlo sampling caused a 2.43% reduction in accuracy. These results demonstrate that each mechanism in the model contributes significantly to the overall performance.

To further examine the role of the graph evolution mechanism under unstructured initial conditions, variants with random adjacency matrix initialization were evaluated. R-GAT (random initialization without the evolution block) achieved a mean accuracy of 47.8%, while R-SFE-GAT (full evolution mechanism applied from the random initialization) reached 59.5%. This corresponds to an average accuracy improvement of 11.8 percentage points when the evolution mechanism is enabled. Similar trends were observed in other metrics: Precision increased by 10.2 points (44.7% to 54.9%), F1-score by 18.7 points (38.3% to 56.9%), and Kappa by 0.225 (0.280 to 0.505).

### 3.6. Visualization Analysis

**Figure 5:**
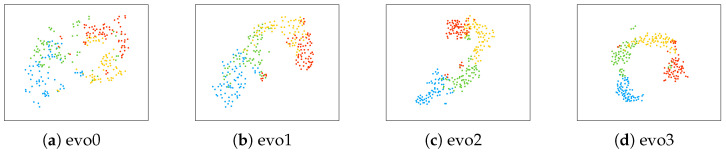
t-SNE visualizations of node features across stages of graph evolution for Subject A01 from the BCI Competition IV-2a dataset. evo0 denotes the initial graph; evo1, evo2, and evo3 denote progressively evolved graphs.

To understand the feature extraction performance more deeply, this study compares the output features of the proposed model with the node features from the initial graph structure and illustrates the feature changes during the progressive graph evolution process. t-SNE, a widely used non-linear dimensionality reduction technique, was employed to project the high-dimensional data into a two-dimensional space, enabling intuitive visualization of the data distribution and structure. Furthermore, the t-SNE results were quantitatively evaluated by calculating the Silhouette Score (Sil Score) and the Davies–Bouldin Index (DBI) of the resulting clusters. Figure 5 displays the visualization results of node features at different graph evolution stages for Subject A01 from the BCI Competition IV Dataset 2a. Table 2 presents a comparison of the average Sil Score and DBI across all subjects’ data at different evolution stages.

The t-SNE visualization in Figure 5 shows the distribution of node features in a two-dimensional space across different graph evolution stages, reflecting the model’s performance in feature extraction and classification. Specifically, Figure 5a shows the distribution of node features under the initial graph topology. It reveals only slight inter-class distinctions, with substantial overlap and mixing of features. Figure 5b–d illustrate the progression of node features during graph evolution, with inter-class separation among the four classes gradually increasing. By the final stage (Figure 5d), the distribution of node features exhibits greater concentration and clear inter-class separation, indicating the model’s ability to extract discriminative, task-relevant features.

The results in Table 2 align with the patterns observed in Figure 5. Relative to the initial graph, the graph-evolved model attains a higher Silhouette Score and a lower Davies–Bouldin Index, indicating improved cluster quality. This indicates that the graph evolution process effectively enhances model performance by making features of different classes more separable and the clustering more distinct.

### 3.7. Brain Network Evolution Analysis

**Figure 6:**
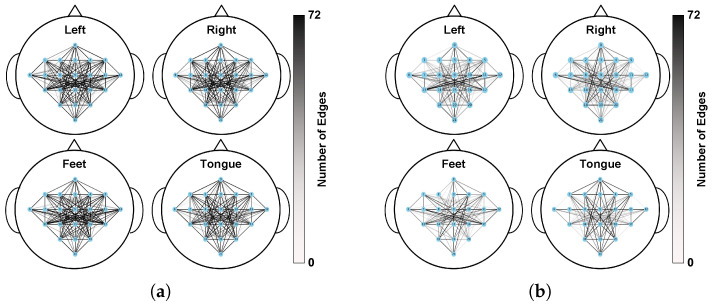
Effect of graph evolution on the adjacency structure: the evolved graph becomes sparser while preserving more informative edges (shown for k = 0 and k = 3). (**a**) Occurrence times of the edges before evolution (k = 0); (**b**) Occurrence times of the edges after evolution (k = 3).

To further explore the mechanism of inter-layer evolution, in the holdout setting, the number of times each edge was retained in the 72 trial sessions of subject A01 was statistically analyzed, and the brain network structures before and after evolution were visually analyzed. Figure 6 shows the distribution of the occurrence times of the edges, with darker colors indicating a higher number of retentions. Before evolution (Figure 6a), the connections were more localized and dense; after evolution (Figure 6b), the graph structure was more sparse, with selection concentrated on a few long-range/inter-regional connections, and the count distributions under different conditions showed differentiated patterns, suggesting that the graph evolution block can adaptively adjust the topology to highlight class-specific connections related to the task.

### 3.8. Performance Comparison

To further validate the performance of the proposed model, a comparative analysis was conducted against several benchmark EEG decoding methods, including EEGNet [39], DGCNN [40], FBCNet [41], EEGConformer [42], LightConvNet [43], IFNet [44], SF-TGCN [12], and MSVTNet [45]. The comparative results in both paradigms are presented in Table 3.

As shown in Table 3, the proposed model demonstrates superior performance under both paradigms. Specifically, under the SD paradigm, the proposed model achieves a mean accuracy of 77.05%, representing improvements of 5.48%, 3.05%, 3.05%, 7.56%, 0.96%, 2.01%, 1.66%, and 1.66% over EEGNet, DGCNN, FBCNet, EEGConformer, LightConvNet, IFNet, SF-TGCN, and MSVTNet, respectively. Furthermore, the proposed model achieves a mean Kappa value of 0.7027, a mean precision of 0.7802, and a mean F1-score of 0.7771, demonstrating a significant advantage.

Under the SI paradigm, the proposed model reaches a mean accuracy of 66.59%, exceeding EEGNet, DGCNN, FBCNet, EEGConformer, LightConvNet, IFNet, SF-TGCN, and MSVTNet by 6.87%, 2.70%, 17.63%, 7.56%, 14.85%, 4.78%, 0.96%, and 2.01%, respectively. Additionally, the proposed model achieves a mean Kappa value of 0.5545, a mean precision of 0.6648, and a mean F1-score of 0.6640, all of which are significantly higher than those of the benchmark models. These results highlight the exceptional capability of the proposed model in decoding EEG signals, consistently achieving high-performance classification of motor imagery tasks. Compared to other benchmark models, the novel architectural design of the proposed model proves more effective in extracting features relevant to motor imagery, thereby confirming its superiority in EEG signal decoding tasks.

In addition to classification performance, inference latency was evaluated using a minimal pure PyTorch implementation. The proposed model achieves an average inference latency of 0.066 ms per sample. This value is competitive with lightweight CNN-based models such as EEGNet (0.014 ms) and LightConvNet (0.021 ms), as well as GNN-based approaches including DGCNN (0.047 ms), FBCNet (0.052 ms), IFNet (0.035 ms), and SF-TGCN (0.072 ms). In contrast, Transformer-based models exhibit substantially higher latency, with EEGConformer at 2.095 ms and MSVTNet at 1.478 ms. These results indicate that the dynamic graph evolution mechanism introduces only moderate computational overhead while maintaining high classification performance.

### 3.9. Cross-Dataset Evaluation

To further assess the robustness and generalization capability of the proposed SFE-GAT, we conducted experiments on the OpenBMI dataset [46]. For fair comparison, we selected the same 22 electrodes as in BCIC-IV-2a and focused on the left-hand and right-hand motor imagery task from Session 1.

The models trained on BCIC-IV-2a were directly evaluated on OpenBMI data in a zero-shot cross-dataset setting. Performance metrics (mean ± standard deviation across subjects) are summarized in Table 4.

## 4. Discussion

This study proposes an inter-layer graph evolution-based neural network model, Structure-Feature Evolution Graph Attention Network (SFE-GAT), for decoding MI-EEG signals. Experimental results demonstrate that the model achieves excellent and robust classification performance under both the SD and SI paradigms. This success is primarily attributed to the model’s structure-feature co-evolution mechanism, which dynamically denoises and optimizes the initial functional brain connectivity graph constructed based on neuroscientific priors, thereby more accurately capturing the spatio-spectral patterns relevant to the motor imagery task.

An effective graph structure is fundamental to the performance of graph neural networks. The initial adjacency matrix in this study was constructed based on the PLV, chosen for its relative insensitivity to signal amplitude, which helps suppress noise in EEG signals. Node features were extracted as the power within frequency bands from 8 to 40 Hz with 4 Hz intervals, designed to cover motor imagery-related rhythms such as the mu and beta bands. However, initial graphs built on fixed priors inevitably contain noise and redundant connections. To address this, the model incorporates a graph evolution module that iteratively refines node representations and graph topology through the alternating collaboration of a GAT and a GAE. The GAE employs a probabilistic edge update strategy based on Bernoulli sampling, which essentially performs approximate posterior sampling of the latent graph structure and can be viewed as a guided form of DropEdge [47]. Ablation experiments (Figure 4) confirmed the critical role of this dynamic structural optimization, as disabling the module led to a significant performance drop. Notably, the Monte Carlo-based edge update strategy employed in the GAE, which models edges as random variables, is less sensitive to hyperparameters than hard-pruning methods and improves the model’s robustness to the inherent uncertainty of EEG signals and inter-subject variability.

The model’s strong overall performance under both SD and SI paradigms (Figure 3) validates its powerful feature extraction and generalization capabilities. It is noteworthy that Subject A02 exhibited suboptimal performance under both paradigms, consistent with the phenomenon of “BCI-illiteracy” reported in the literature, where some subjects struggle to generate stable motor imagery EEG patterns. This finding underscores the need for future research to focus on enhancing model adaptability to individual differences. The proposed graph-evolution mechanism may offer a promising direction for mitigating BCI illiteracy. By dynamically refining functional connectivity through inter-layer co-adaptation, SFE-GAT could potentially be combined with transfer learning or domain-adaptation strategies to better accommodate inter-subject variability and improve performance for low-performing users.

Ablation studies systematically validated the contribution of each component. Replacing the GAT with a GCN resulted in performance degradation, highlighting the advantage of the attention mechanism in adaptively weighting node importance [48]. Furthermore, removing the Monte Carlo-based edge evolution mechanism also led to a performance decrease, albeit to a lesser extent, indicating that the introduced probabilistic regularization effectively enhances model robustness. Additionally, even when starting from a completely random adjacency matrix, enabling the graph evolution mechanism produced consistent improvements across multiple evaluation metrics compared to the non-evolutionary baseline. This outcome further supports the mechanism’s ability to adaptively learn and refine task-relevant functional connectivity, demonstrating its significant contribution even in the absence of structured initial priors.

The efficiency of the proposed model is further supported by its inference latency. As shown in Table 3, The model exhibits highly competitive latency compared to lightweight CNN-based and GNN-based baselines, while being substantially faster than Transformer-based approaches. These observations indicate that the dynamic graph evolution mechanism introduces only moderate computational overhead, preserving real-time feasibility without compromising classification performance. This efficiency advantage highlights the practical value of graph-based dynamic architectures over attention-heavy Transformer models for resource-constrained EEG decoding applications.

Visualization of the feature embeddings and cluster evaluation provided intuitive support for the effectiveness of the structure-feature co-evolution. As shown in Figure 5, after three evolution stages, the Sil Score increased monotonically from 0.3088 to 0.3992, while the DBI decreased from 0.9427 to 0.7909. This trend clearly indicates that the evolution process significantly enhances intra-class compactness and inter-class separation.

The cross-dataset evaluation on OpenBMI further strengthens this conclusion. As shown in Table 4, despite the domain shift and higher inter-subject variability compared to BCIC-IV-2a, the proposed model maintained competitive performance in the binary motor imagery task. These observed results demonstrate that the graph evolution mechanism captures generalizable EEG connectivity patterns across different datasets, acquisition systems, and task formulations.

The model’s enhanced performance stems primarily from its ability to effectively simulate the brain’s adaptive optimization of functional networks during motor imagery through a structure–feature evolution mechanism. Visualization of brain network evolution reveals that, as network depth increases, node features gradually form more distinct class boundaries, illustrating a shift in neural information processing from sensory representation to abstract intention encoding. Moreover, deeper network layers tend to preserve a small subset of high-frequency, critical connections. This pattern aligns with neuroscientific observations commonly referred to as the “task core network” or “efficient information routing,” in which the brain strengthens information transmission along a limited number of key pathways during task execution.

In summary, the proposed inter-layer graph evolution model provides an effective solution for MI-EEG decoding through dynamic graph structure optimization. Comparative results with existing benchmark methods (Table 3) further confirm the competitive advantage of the proposed model. However, this study has certain limitations. First, model performance remains somewhat influenced by individual differences, as evidenced by the results for Subject A02. Second, the initial graph structure relies on PLV; future work could explore other connectivity metrics or data-driven initialization methods. Future efforts will focus on developing more efficient graph evolution strategies and exploring the application potential of this framework to a wider range of EEG paradigms, such as emotion recognition and sleep stage scoring, to advance graph neural networks in the field of EEG decoding.

## 5. Conclusions

To overcome the representational constraints of static graphs in motor imagery EEG decoding, this study introduces SFE-GAT, a graph neural network founded on dynamic graph structure learning. The principal innovation is an inter-layer graph evolution mechanism, engineered to emulate the dynamic and hierarchical reorganization of functional brain connectivity throughout motor imagery tasks. The proposed model employs neurophysiologically interpretable features, which include functional connectivity based on phase-locking value and multiband power spectral density used as nodal attributes. It leverages graph attention networks and graph autoencoders to enable the co-adaptation of graph topology and node features throughout the network layers.

Experiments conducted on the BCI Competition IV-2a dataset show that the proposed method significantly enhances decoding performance in multiple motor imagery tasks, consistently surpassing state-of-the-art baselines across different evaluation frameworks. The evolved graph structures display sparsification and strengthening of task-relevant connections, intuitively mirroring the hierarchical information routing patterns of the brain during cognitive processing. Consequently, this study not only delivers an effective dynamic graph optimization tool for MI-EEG decoding but also introduces a novel computational perspective for investigating the hierarchical dynamic representations involved in motor intention encoding. This approach forges a meaningful connection between adaptive graph learning and neural dynamics, offering valuable insights for developing next-generation brain–computer interface systems and underscoring the potential of computational models in elucidating neural mechanisms.

## Figures and Tables

**Table 1:** Model architecture and parameters.

Blocks	Layers	Input Channels	Hidden Channels	Output Channels	Heads	Parameters
	GATv2Conv	8	32	128	4	2560
GraphEvoBlock-1	GATv2Conv	128	32	128	4	33,280
	BatchNorm	128	128	128	-	256
	GAE	128	32	32	-	4192
	MC-EdgeGen	-	-	-	-	-
GraphEvoBlock-2	GATv2Conv	128	32	128	4	33,280
	BatchNorm	128	128	128	-	256
	GAE	128	32	32	-	4192
	MC-EdgeGen	-	-	-	-	-
GraphEvoBlock-3	GATv2Conv	128	32	128	4	33,280
	BatchNorm	128	128	128	-	256
	GAE	128	32	32	-	4192
	MC-EdgeGen	-	-	-	-	-
Classifier	GATv2Conv	128	32	128	4	33,280
	BatchNorm	128	128	128	-	256
	GlobalMeanPool	128	-	128	-	-
	Dropout	128	-	128	-	-
	Linear	128	4	4	-	516

**Table 2:** Comparison Results of average Silhouette Score (Sil Score) AND Davies–Bouldin Index (DBI).

	evo0	evo1	evo2	evo3
Sil Score	0.3088	0.3264	0.3797	0.3992
DBI	0.9427	0.8855	0.8114	0.7909

**Table 3:** Comparative Performance Metrics in Subject-Dependent and Subject-Independent Analyses on the BCIC-IV-2A Dataset.

Model	Subject-Dependent				Subject-Independent				Inference Latency
	Accuracy	Kappa	Precision	F1-Score	Accuracy	Kappa	Precision	F1-Score	(ms/Sample)
EEGNet (2018)	0.7222	0.6243	0.7433	0.7182	0.5972	0.4591	0.6471	0.5850	**0.014**
DGCNN (2020)	0.7465	0.6497	0.7612	0.7461	0.6389	0.5069	0.6862	0.6289	0.047
FBCNet (2020)	0.7465	0.6535	**0.7819**	0.7396	0.4896	0.3140	0.5490	0.4475	0.052
EEGConformer (2022)	0.7014	0.5524	0.7075	0.6996	0.5903	0.4455	0.6162	0.5823	2.095
LightConvNet (2022)	0.7674	0.6790	0.7891	0.7509	0.5174	0.3460	0.5968	0.4893	0.021
IFNet (2023)	0.7569	0.6626	0.7654	0.7560	0.6181	0.4817	**0.6927**	0.6045	0.035
SF-TGCN (2024)	0.7604	0.6628	0.7743	0.7592	0.6563	0.5239	0.7032	0.6459	0.072
MSVTNet (2024)	0.7604	0.6641	0.7808	0.7490	0.6458	0.5072	0.7012	0.6317	1.478
Proposed (2025)	**0.7770**	**0.7027**	0.7802	**0.7771**	**0.6659**	**0.5545**	0.6648	**0.6640**	0.066

**Table 4:** Performance on OpenBMI Binary Motor Imagery Task (left vs. right hand, Session 1). Mean ± standard deviation across subjects.

Model	Accuracy (%)	Kappa
EEGNet	68.51 ± 12.7	0.52 ± 0.15
DGCNN	65.49 ± 13.1	0.48 ± 0.17
FBCNet	64.57 ± 13.5	0.47 ± 0.16
EEGConformer	58.18 ± 14.2	0.40 ± 0.18
LightConvNet	69.48 ± 12.4	0.53 ± 0.14
IFNet	66.28 ± 13.3	0.49 ± 0.16
SF-TGCN	64.54 ± 13.5	0.47 ± 0.17
MSVTNet	59.56 ± 15.5	0.42 ± 0.19
**Proposed**	**67.18 ± 13.6**	**0.51 ± 0.16**

## Data Availability

The data that support the findings of this study are available upon reasonable request from the authors. The source code, trained models, and preprocessing scripts are publicly available at https://github.com/GaoHSin/SFE-GAT (12 January 2026) to facilitate reproducibility and independent evaluation.

## Abbreviations

The following abbreviations are used in this manuscript:

EEG	Electroencephalography
BCI	Brain–Computer Interface
MI	Motor imagery
GNN	Graph Neural Network
PLV	Phase-Locking Value
PSD	Power Spectral Density
GAT	Graph Attention Network
GAE	Graph Autoencoder
GCN	Graph Convolutional Network
SD	Subject-dependent
SI	Subject-independent
DBI	Davies–Bouldin Index
